# Behavior of the Energy Spectrum and Electric Conduction of Doped Graphene

**DOI:** 10.3390/ma13071718

**Published:** 2020-04-06

**Authors:** Stefano Bellucci, Sergei Kruchinin, Stanislav P. Repetsky, Iryna G. Vyshyvana, Ruslan Melnyk

**Affiliations:** 1 INFN-Laboratori Nazionali di Frascati, 40 Via E. Fermi, 00044 Frascati, Italy; 2 Bogolyubov Institute for Theoretical Physics, NASU, Metrolohichna Str. 14-b, 03143 Kyiv, Ukraine; sergeikruchinin@yahoo.com; 3 Institute of High Technologies, Taras Shevchenko National University of Kiev, 4-g, Academician Glushkov Ave., 03022 Kiev, Ukraine; i.vyshyvana@gmail.com; 4 Physical and Mathematical Sciences, National University “Kyevo-Mohylyans’ka Akademiya”, 2 G. Skovoroda Str., 04070 Kyiv, Ukraine; rmelnyk@ukr.net

**Keywords:** graphene, energy gap, density of states, electrical conductance, impurity concentration, ordering parameter, metal–insulator transition

## Abstract

We consider the effect of atomic impurities on the energy spectrum and electrical conductance of graphene. As is known, the ordering of atomic impurities at the nodes of a crystal lattice modifies the graphene spectrum of energy, yielding a gap in it. Assuming a Fermi level within the gap domain, the electrical conductance diverges at the ordering of graphene. Hence, we can conclude about the presence of a metal–dielectric transition. On the other hand, for a Fermi level occurring outside of the gap, we see an increase in the electrical conductance as a function of the order parameter. The analytic formulas obtained in the Lifshitz one-electron strong-coupling model, describing the one-electron states of graphene doped with substitutional impurity atoms in the limiting case of weak scattering, are compared to the results of numerical calculations. To determine the dependence of the energy spectrum and electrical conductance on the order parameter, we consider both the limiting case of weak scattering and the case of finite scattering potential. The contributions of the scattering of electrons on a vapor of atoms to the density of states and the electrical conductance of graphene with an admixture of interstitial atoms are studied within numerical methods. It is shown that an increase in the electrical conductance with the order parameter is a result of both the growth of the density of states at the Fermi level and the time of relaxation of electron states. We have demonstrated the presence of a domain of localized extrinsic states on the edges of the energy gap arising at the ordering of atoms of the admixture. If the Fermi level falls in the indicated spectral regions, the electrical conductance of graphene is significantly affected by the scattering of electrons on clusters of two or more atoms, and the approximation of coherent potential fails in this case.

## 1. Introduction

Modifications of graphene with impurities, defects, and chemical functional groups are of increasing interest, as they change the physical properties of graphene and make it a basic system, generating a new class of functional materials. Such materials can be supposedly used in nanoelectromechanical systems, systems of accumulation of hydrogen, etc. as competitors of silicon in electronic devices, though the quasirelativistic spectrum of charge carriers in graphene hampers its application, for example, in field transistors due to the absence of a gap in its spectrum. However, it is worth noting that the impurities can induce the appearance of such a gap.

Indeed, within the self-consistent meta-gradient approximation and the method of projection of adjoint waves [1], the numerical calculations executed by this method showed the opening of a gap in the energy spectrum of graphene due to the presence of an impurity. The electronic structures of the isolated monolayer of graphene, two- and three-layer graphene, and graphene grown on ultrathin layers of hexagonal boron nitride (h-BN) were calculated in [2] in the frame of the pseudopotential method. It was shown that a forbidden energy band 57 meV in width appears in graphene grown on a monolayer of h-BN.

Similar calculations for graphene with impurities of aluminum, silicon, phosphorus, sulfur, boron, nitrogen, and lithium were executed in works [3,4], where the opening of a gap was demonstrated, in particular. 

It is clear that the numerical calculations should be supported by a simple but adequate model presenting the exact analytic solutions. The theory of the modification of the spectrum of graphene caused by an increase in the concentration of point impurities was developed in works [5,6,7], where the possibility of the metal–dielectric transition was predicted, and the dominant role of a quasigap filled by localized states in the scattering by pairs and triples of impurity centers was indicated. 

The Kubo–Greenwood quantum-mechanical formalism in the Lifshitz one-band model was applied in [8,9,10,11,12,13] to study the influence of impurity atoms or atoms adsorbed on the surface on the electronic structure and electrical conductance of graphene. There, the method of the reduction of the Hamiltonian to the three-diagonal form was developed to study the influence of completely ordered impurity atoms on the energy spectrum and electrical conductance of graphene. In work [10], it was found that a gap of 0.45 eV in width appears in the energy spectrum of electrons of graphene deposited on a potassium substrate. It was assumed that the appearance of this gap is associated with a change in the symmetry of the crystal. This assumption was confirmed in work [14], where the influence of the atomic ordering on the energy spectrum and electrical conductance of an alloy was analytically studied. It was established [14] that, for a long-range ordering of the alloy, a gap arises in the energy spectrum of electrons. It was also found that, in the case where the Fermi level falls in the domain of the gap and at a long-range atomic ordering, a metal–dielectric transition appears in the alloy.

We note that, having the Fermi level falling in the gap domain, the velocity of an electron can lower its speed on such a level. This leads to a decrease in the mobility of electrons and in the electrical conductance, which can worsen the functional characteristics of graphene.

Introducing the notation η as the ordering parameter, δ. as the difference in the scattering potentials of impurity atoms and carbon, and y as the impurity concentration, it was established in Ref. [15] that a gap with width $\eta \left|\delta \right|$ and positioned at point yδ arises in the graphene energy spectrum, as a consequence of the ordering of substitutional atoms at nodes of the crystal lattice. Assuming a Fermi level within the gap domain, at the ordering of graphene, the electrical conductance diverges; hence, we can conclude that we are in the presence of a metal–dielectric transition. On the other hand, for a Fermi level occurring outside of the gap, we see an increase in the electrical conductance as a function of the order parameter η. As $\left(\eta \to 1\right)$ at the concentration y=1/2, the electrical conductance of graphene $\sigma_{\alpha \alpha }\to \infty$, i.e., graphene transits into the state of ideal conductance.

It is worth indicating the different nature of a gap in the energy spectrum of graphene related to edge effects. Work [16] presents an experimental study of the appearance of the energy gap in armchair-like graphene nanoribbons, which increases as the nanoribbon width decreases. In works [17,18] in the model of strong coupling, selection rules for optical transitions between the valence and the conductance zones were determined for armchair and zigzag graphene nanoribbons. Electron and optical properties of inhomogeneous two-layer graphene nanoribbons in the external magnetic field were considered in [19]. Based on the results of numerical calculations, it was shown there that the electron and optical properties of nanoribbons depend strongly on the competition between the magnetic quantization, width of nanoribbons, and arrangement configuration of such nanoribbons. Work [20] gives a review of the electron and optical properties of graphene nanoribbons in magnetic and electric fields with regard to the effects of side limitation, curvature, and structural inhomogeneities. The theoretically predicted parabolic subzones, edge-localized states, opening of a gap, and Landau’s subzones were identified by various experimental measurements. 

In recent times, several tens of works have been published on the influence of various types of deformations on the electron properties of (mainly, defectless) graphene (see, e.g., review [21]). The results of the first calculations within density functional theory [22] asserted that even small deformations can cause the appearance of a forbidden zone. The calculations in the model of strong coupling and linear elasticity theory [23] showed that the forbidden zone appears only at the tensile deformations (~23%) close to the rupture deformation (≈27%). Thus, many questions remain open and require subsequent discussion.

The goal of the present work is to clarify the nature of the influence of the ordering of a substitutional impurity on the appearance of a gap in the energy spectrum and on the electric conductance of graphene.

The conclusions in work [15] were based on the results of analytical studies of the energy spectrum and electrical conductance of graphene performed in the approximation of the coherent potential. However, the domain of convergence of the cluster expansion used in [15] for a Green’s function and the domain of validity of the approximation of the coherent potential were not analyzed. 

In the present work, we will consider the interconnection between a change in the electrical conductance, changes in the energy spectrum, and the decay time for electron states under the ordering of impurity atoms in graphene.

## 2. Theoretical Model

The Hamiltonian in the Lifshitz one-electron strong-coupling model describing the one-electron states of graphene doped with substitutional impurity atoms can be presented in the following form [15]:

(1) $$H=\sum_{ni}|ni\rangle v_{ni}\langle ni|+\sum_{ni,n^{\prime }i^{\prime }\neq ni}|ni\rangle h_{ni,n^{\prime }i^{\prime }}\langle n^{\prime }i^{\prime }|,$$

where $h_{ni,n^{\prime }i^{\prime }}$ is a nondiagonal matrix element of the Hamiltonian (hopping integral) in the Wannier representation and is independent of the random arrangement of atoms in the approximation of diagonal disorder; $\nu_{ni}$ is a diagonal matrix element taking the values $\nu^{A}$ or $\nu^{B}$ depending on that which the atoms A or B is placed at node ni; n is the number of an elementary cell, and i is the number of a node of the sublattice in the elementary cell.

Let us add to and subtract the translationally invariant operator $\sum_{ni}|ni\rangle \sigma_{i}\langle ni|$ from Equation (1), where $\sigma_{i}$ is a diagonal matrix element of the Hamiltonian of some effective ordered medium (coherent potential), depending on the sublattice number. As a result, the Hamiltonian of graphene reads

(2) $$\begin{array}{c} H=\tilde{H}+\tilde{V}\\ \tilde{H}=\underset{ni}{\sum }|ni\rangle \sigma_{i}\langle ni|+\underset{ni,n^{\prime }i^{\prime }\neq ni}{\sum }|ni\rangle h_{ni,n^{\prime }i^{\prime }}\langle n^{\prime }i^{\prime }|\\ \tilde{V}=\sum_{ni}{\tilde{v}}_{ni},{\tilde{v}}_{ni}=|ni\rangle \left(v_{ni}-\sigma_{i}\right)\langle ni|.. \end{array}$$

The formula for the coherent potential will be given below.

The retarded Green’s function of graphene, which is an analytic function in the upper half-plane of values of the complex energy *z*, is defined as

(3) $$G\left(z\right)={\left(z-H\right)}^{-1}.$$

A Green’s function satisfies the equation

(4) $$G=\tilde{G}+\tilde{G}T\tilde{G},$$

where

(5) $$\tilde{G}={\left(z-\tilde{H}\right)}^{-1},$$

is the Green’s function of the effective medium corresponding to the Hamiltonian $\tilde{H}$ in Equation (2). The scattering *T*-matrix can be presented in the form of an infinite series [15]:

(6) $$T=\sum_{\left(n_{1}i_{1}\right)}t^{n_{1}i_{1}}+\sum_{\left(n_{1}i_{1}\right)\neq \left(n_{2}i_{2}\right)}T^{\left(2\right)\;n_{1}i_{1},n_{2}i_{2}}+\ldots .$$

Here,

(7) $$T^{\left(2\right)\;n_{1}i_{1},n_{2}i_{2}}={\left[I-t^{n_{1}i_{1}}\tilde{G}t^{n_{2}i_{2}}\tilde{G}\right]}^{-1}t^{n_{1}i_{1}}\tilde{G}t^{n_{2}i_{2}}\left[I+\tilde{G}t^{n_{1}i_{1}}\right],$$

where

(8) $$t^{n_{1}i_{1}}={\left[I,-,{\tilde{v}}_{in},\tilde{G}\right]}^{-1}{\tilde{v}}_{in}$$

is the operator of scattering on one node, and *I* is the identity operator. 

The terms of Equation (6) describe the processes of multiple scattering of electrons on clusters comprising one, two, three, etc. scattering centers. 

In work [15], it was shown that the contributions of the processes of the scattering of electrons on clusters to the density of states and to the electrical conductance decrease and are guided by some small parameter $\gamma_{i}\left(\varepsilon \right)$ [24], as the number of atoms in a cluster increases. The formula for $\gamma_{i}\left(\varepsilon \right)$ will be presented in the following.

Neglecting the contributions of the processes of scattering on clusters of three or more atoms, which are small due to the parameter $\gamma_{i}\left(\varepsilon \right)$, we give the density of one-electron states of graphene in the following form [15]:

(9) gε=1v∑i,λPλ0igλ0iεgλ0iε=−2π Im G˜+G˜ tλ0iG˜+∑lj≠0iλ′Pλ′lj/λ0i××G˜ tλ′lj+T2λ 0i,λ′ljG˜0i,0i

where ν=2 is the number of sublattices of graphene.

Using the Kubo–Greenwood formula [25] and neglecting the contribution of the processes of scattering on clusters of three and more atoms, we write the static electrical conductance of graphene (at *T* = 0) as [15]

(10) $$\begin{array}{l} \sigma_{\alpha \beta }=-\frac{e^{2}\hbar }{2\pi \Omega_{1}}\underset{s,s^{\prime }=+,-}{\sum }\left(2\delta_{ss^{\prime }}-1\right)\underset{i}{\sum }\left\{\left[v_{\beta }\tilde{K}\left(\varepsilon_{}^{s},v_{\alpha },\varepsilon_{}^{s^{\prime }}\right)\right]+\right.\\ +\underset{\lambda }{\sum }P_{}^{\lambda 0i}\tilde{K}\left(\varepsilon^{s^{\prime }},v_{\beta },\varepsilon^{s}\right)(t_{}^{\lambda 0i}\left(\varepsilon^{s}\right)\tilde{K}\left(\varepsilon^{s},v_{\alpha },\varepsilon^{s^{\prime }}\right)t_{}^{\lambda 0i}\left(\varepsilon^{s^{\prime }}\right)+\\ +\overset{\lambda }{\underset{\lambda }{\sum }}P_{}^{\lambda 0i}\underset{\begin{array}{c} lj\neq 0i,\\ \lambda^{\prime } \end{array}}{\sum }P_{}^{\lambda^{\prime }lj/\lambda 0i}\left[[\tilde{K}\left(\varepsilon^{s^{\prime }},v_{\beta },\varepsilon^{s}\right)v_{\alpha }\tilde{G}\left(\varepsilon^{s^{\prime }}\right)\right.]\times \\ \times T^{\left(2\right)\lambda 0i,\lambda^{\prime }lj}\left(\varepsilon^{s^{\prime }}\right)+\\ +\left[\tilde{K}\left(\varepsilon^{s},v_{\alpha },\varepsilon^{s^{\prime }}\right)v_{\beta }\tilde{G}\left(\varepsilon^{s}\right)\right]T^{\left(2\right)\lambda \;0i,\lambda^{\prime }lj}\left(\varepsilon^{s}\right)+\\ +\tilde{K}\left(\varepsilon^{s^{\prime }},v_{\beta },\varepsilon^{s}\right)\left[t_{}^{\lambda^{\prime }lj}\left(\varepsilon^{s}\right)\tilde{K}\left(\varepsilon^{s},v_{\alpha },\varepsilon^{s^{\prime }}\right)t_{}^{\lambda 0i}\left(\varepsilon^{s^{\prime }}\right)\right.+\\ +\left(t_{0i}^{\lambda }\left(\varepsilon^{s}\right)+t_{lj}^{\lambda^{\prime }}\left(\varepsilon^{s}\right)\right)\tilde{K}\left(\varepsilon^{s},v_{\alpha },\varepsilon^{s^{\prime }}\right)T^{\left(2\right)\lambda 0i,\lambda^{\prime }lj}\left(\varepsilon^{s^{\prime }}\right)+\\ +T^{\left(2\right)\lambda^{\prime }lj,\lambda \;0i}\left(\varepsilon^{s}\right)\tilde{K}\left(\varepsilon^{s},v_{\alpha },\varepsilon^{s^{\prime }}\right)t_{}^{\lambda 0i}\left(\varepsilon^{s^{\prime }}\right)+\\ +T^{\left(2\right)\lambda^{\prime }lj,\lambda \;0i}\left(\varepsilon^{s}\right)\tilde{K}\left(\varepsilon^{s},v_{\alpha },\varepsilon^{s^{\prime }}\right)T^{\left(2\right)\lambda \;0i,\lambda^{\prime }lj}\left(\varepsilon^{s^{\prime }}\right)+\\ +{\left.{\left.\left.\left.T^{\left(2\right)\lambda^{\prime }lj,\lambda \;0i}\left(\varepsilon^{s}\right)\tilde{K}\left(\varepsilon^{s},v_{\alpha },\varepsilon^{s^{\prime }}\right)T^{\left(2\right)\lambda^{\prime }lj,\lambda \;0i}\left(\varepsilon^{s^{\prime }}\right)\right]\right]\right\}}_{0i,0i}\right|}_{\varepsilon =\mu }. \end{array}$$

$\tilde{K}\left(\varepsilon_{}^{s},v_{\alpha },\varepsilon_{}^{s^{\prime }}\right)=\tilde{G}\left(\varepsilon_{}^{s}\right)v_{\alpha }\tilde{G}\left(\varepsilon_{}^{s^{\prime }}\right)$, $\tilde{G}\left(\varepsilon_{}^{+}\right)={\tilde{G}}_{r}\left(\varepsilon \right)$, $\tilde{G}\left(\varepsilon_{1}^{-}\right)={\tilde{G}}_{a}\left(\varepsilon \right)={\left({\tilde{G}}_{r}\right)}^{*}\left(\varepsilon \right)$, and ${\tilde{G}}_{r}\left(\varepsilon \right)$ and ${\tilde{G}}_{a}\left(\varepsilon \right)$ are the retarded and advanced Green’s functions, respectively; $\Omega_{1}=2\Omega_{0}$ is the volume of an elementary cell of graphene, $\Omega_{0}$ is the volume per atom, ℏ is Planck’s constant, and

(11) $${\tilde{G}}_{njn^{\prime }j^{\prime }}\left(\varepsilon \right)=\frac{1}{N}\underset{k}{\sum }{\tilde{G}}_{jj^{\prime }}\left(k,\varepsilon \right)exp(ik\left(r_{n^{\prime }j^{\prime }}-r_{nj}\right))$$

where ${\tilde{G}}_{jj^{\prime }}\left(k,\varepsilon \right)$ is the Fourier transform of the Green’s function of an effective medium, and $r_{nj}$ is the radius-vector of node nj. The wave vector k varies in the limits of the Brillouin zone of graphene.

The operator of the electron velocity projection α is given by

(12) $$\upsilon_{\alpha ii’}\left(k\right)=\frac{1}{\hbar }\frac{\partial h_{ii\prime }\left(k\right)}{\partial k_{\alpha }},$$

where $h_{jj^{\prime }}\left(k\right)$ is the Fourier transform of the hopping integral. We calculate $h_{ii\prime }\left(k\right)$ in the approximation of the nearest neighbors:

(13) $$h_{jj^{\prime }}\left(k\right)=h_{1}\sum_{n^{\prime }\neq n}exp(ik\left(r_{n^{\prime }j^{\prime }}-r_{nj}\right)),$$

where $h_{1}=\left(pp\pi \right)$ is the hopping integral [26] and $r_{nj}$ is the radius-vector of node jn.

The Fermi level *μ* is determined from the relation

(14) $$\langle Z\rangle =\int_{-\infty }^{\mu }g\left(\varepsilon \right)\;d\varepsilon ,$$

where 〈Z〉 is the mean number of electrons per atom whose energies belong to the energy zone.

In Equations (9) and (10), $P_{}^{\lambda 0i}$ is the probability of the filling of node 0i of the crystal sublattice i=1,2 by atoms of the sort λ=A,B. We have

(15) $$P^{B01}=y_{1}=y+\frac{1}{2}\eta ,P^{B02}=y_{2}=y-\frac{1}{2}\eta ,P^{A0i}=1-P^{B0i},$$

where y is the concentration of impurity atoms and η is the long-range atomic order parameter.

In Equations (9) and (10), $P_{}^{\lambda^{\prime }lj/\lambda 0i}$ is the probability of the filling of node lj by atoms of the sort λ′ under the condition that an atom of the sort λ occupies node 0i (the parameter of binary interatomic correlations in the filling of nodes of the crystal lattice by impurity atoms). 

The probabilities are determined by the interatomic pair correlations $\varepsilon_{lj0i}^{BB}$ via [27,28]

(16) $$P_{lj0i}^{\lambda \prime /\lambda }=P_{lj}^{\lambda \prime }+\frac{\varepsilon_{lj0i}^{BB}}{P_{0i}^{\lambda }}\left(\delta_{\lambda \prime B}-\delta_{\lambda \prime A}\right)\left(\delta_{\lambda B}-\delta_{\lambda A}\right),$$

where δ is the Kronecker delta function. Note that the interatomic pair correlations also satisfy the relation

(17) $$\varepsilon_{lj0i}^{BB}=\langle \left(c_{lj}^{B}-c_{j}^{B}\right)\left(c_{0i}^{B}-c_{i}^{B}\right)\rangle .$$

Here, $c_{lj}^{B}$ is a random number equal to 1, if an atom of the sort B occupies node lj, or to zero in the opposite case; ${с}_{j}^{B}=\langle c_{0j}^{B}\rangle =P^{B0j}$. The brackets mean the averaging over the distribution of impurity atoms at nodes of the crystal lattice.

The coherent potential can be determined from the condition $\langle t^{n_{1}i_{1}}\rangle =0$, which yields the equation [15]

(18) $$\sigma_{i}=\langle \upsilon_{i}\rangle -\left(\upsilon_{A}-\sigma_{i}\right){\tilde{G}}_{0i,0i}\left(\varepsilon \right)\left(\upsilon_{B}-\sigma_{i}\right);\;\langle \upsilon_{i}\rangle =\left(1-y_{i}\right)\upsilon_{A}+y_{i}\upsilon_{B}.$$

Setting $\upsilon_{A}=0$, we obtain

(19) $$\langle \upsilon_{i}\rangle =y_{i}\delta ,$$

where

(20) $$\delta =\upsilon_{B}-\upsilon_{A},$$

is the difference of the scattering potentials for the components of graphene.

Solving the system of Equations (11) and (18), we find the values of the coherent potential $\sigma_{i}$.

In the limiting case of weak scattering $\left|\delta /w\right|\ll 1$, where $w=3\left|h_{1}\right|$ is the half-width of the energy zone of pure graphene, the given theoretical model admits analytic solutions [14]. It was established that the arising gap $\eta \left|\delta \right|$ in width is centered at the point yδ.

Equation (15) implies that the maximum order parameter $\eta_{max}$. For δ>0 and δ<0, the gap is placed, respectively, to the right and left of the Dirac point on the energy scale.

At the electron concentration 〈Z〉 (Equation (14)), at which the Fermi level falls in the arising gap, the electrical conductance tends to zero at the ordering of an impurity, $\sigma_{\alpha \alpha }\to 0$, i.e., a metal–dielectric transition occurs [15].

At the electron concentration 〈Z〉 (Equation (14)) such that the Fermi level is outside the arising gap, the electrical conductance increases with the order parameter η [15]. As the order parameter η tends to its maximum value $\eta_{max}$, the electrical conductance diverges, $\sigma_{\alpha \alpha }\to \infty$, i.e., graphene transits in the state of ideal conductance.

We recall that the contributions of the processes of the scattering of electrons on clusters to the density of states and to the electrical conductance decrease and are guided by some small parameter $\gamma_{i}\left(\varepsilon \right)$ [24], as the number of atoms in a cluster increases. This parameter is

(21) $$\gamma_{i}\left(\varepsilon \right)=\left|\langle {\left(t^{oi}\left(\varepsilon \right)\right)}^{2}\rangle \underset{lj\neq 0i}{\sum }{\tilde{G}}_{oi,lj}\left(\varepsilon \right){\tilde{G}}_{lj,oi}\left(\varepsilon \right)\right|\;\langle {\left(t^{oi}\left(\varepsilon \right)\right)}^{2}\rangle =\left(1-y_{i}\right){\left({t^{A}}^{oi}\left(\varepsilon \right)\right)}^{2}+y_{i}{\left({t^{B}}^{oi}\left(\varepsilon \right)\right)}^{2}.$$

The parameter $\gamma_{i}\left(\varepsilon \right)$ can be represented in the form [14]

(22) $$\begin{array}{l} \gamma_{i}\left(\varepsilon \right)=\left|P_{i}\left(\varepsilon \right)/\left(1+P_{i}\left(\varepsilon \right)\right)\right|\\ P_{i}\left(\varepsilon \right)=-\frac{\langle {\left(t^{0i}\left(\varepsilon \right)\right)}^{2}\rangle }{1+\langle {\left(t^{0i}\left(\varepsilon \right)\right)}^{2}\rangle {\left({\tilde{G}}_{0i,0i}\left(\varepsilon \right)\right)}^{2}}\left(\frac{1}{1+\langle {\left(t^{0i}\left(\varepsilon \right)\right)}^{2}\rangle {\left({\tilde{G}}_{0i,0i}\left(\varepsilon \right)\right)}^{2}}\frac{d}{d\varepsilon }{\tilde{G}}_{0i,0i}\left(\varepsilon \right)+{\left({\tilde{G}}_{0i,0i}\left(\varepsilon \right)\right)}^{2}\right) \end{array}$$

Using formula ${\tilde{G}}_{0i,0i}\left(\varepsilon \right)$ obtained in [14] in the limiting case of weak scattering $\left|\delta /w\right|\ll 1$, it can be shown that the parameter $\gamma_{i}\left(\varepsilon \right)$ takes values $1/2\leq \gamma_{i}\left(\varepsilon \right)\leq 1$ in a narrow interval of energies in the energy gap:

(23) $$\left|\frac{\Delta \varepsilon^{\prime }\left(\eta \right)}{w}\right|=\frac{27}{\pi }\left(y^{2}-\frac{1}{4}\eta^{2}\right)((1-y{)}^{2}-\frac{1}{4}\eta^{2})\eta {\left(\frac{\delta }{w}\right)}^{5}.$$

The parameter $\gamma_{i}\left(\varepsilon \right)$ is small except for the narrow energy intervals (Equation (23)) on the gap edges. As the value of the energy tends to the gap edge, $d{\tilde{G}}_{0i,0i}\left(\varepsilon \right)/d\varepsilon \to \infty$ [14], whereas the parameter $\gamma_{i}\left(\varepsilon \right)\to 1$ (Equation (22)).

Thus, the processes of scattering on clusters give a significant contribution to the density of states at the energies of electrons lying in interval (Equation (23)). The product *σ_xx_*·*d* enters the formula for the electrical resistance of a graphene layer

(24) $$R=\frac{1}{\sigma_{xx}d}\frac{l}{L},$$

where *l* is the length of a graphene layer along the axis *x*, and *L* is the layer width. Here, d is the thickness of graphene. The axis *x* is directed from a carbon atom to its nearest neighbor. The values of *σ_xx_*·*d* are given in units of $e^{2}\cdot \hbar^{-1}$.

To establish the nature of the dependence of the electrical conductivity $\sigma_{\alpha \alpha }$ on the order parameter *η*, we consider both the limiting case of weak scattering, $\left|\delta /w\right|\ll 1$, and the case of a finite value of the scattering potential δ/w.

If the order parameter *η* tends to its maximum value, $\eta \to \eta_{max}$, the nodes of sublattice 2 are mainly occupied by carbon atoms. In this case, Equations (15) and (18) imply that the imaginary part of the coherent potential for this sublattice tends to zero, $\sigma_{2}^{\prime \prime }\left(\mu \right)\to 0$, and the formula for the electrical conductance (Equation (10)) in the approximation of coherent potential takes the form

(25) $$\sigma_{\alpha \alpha }=\frac{e^{2}\hbar }{3\Omega_{0}}\frac{g_{2}\left(\mu \right)\;|\upsilon_{\alpha 12}\left(\mu \right){|}^{2}}{\left|\sigma_{2}^{\prime \prime }\left(\mu \right)\right|},$$

where $g_{2}\left(\mu \right)$ is the partial density of states for the second sublattice.

For the three-dimensional crystals with a simple lattice in the approximation of effective mass, Equation (25) with the corresponding $g\left(\mu \right)$ and $\upsilon_{\alpha }\left(\mu \right)$ takes the well-known form

(26) $$\sigma_{\alpha \alpha }=e^{2}n\tau \left(\mu \right)/m^{*},$$

where n is the number of electrons in unit volume with energies less than the Fermi level, $m^{*}$ is the effective mass of an electron, and $\tau \left(\mu \right)$ is the relaxation time of electron states, which is defined by the relation

(27) $$\left|\sigma_{}^{\prime \prime }\left(\mu \right)\right|\tau \left(\mu \right)=\hbar .$$

## 3. Results

**Figure 1:**
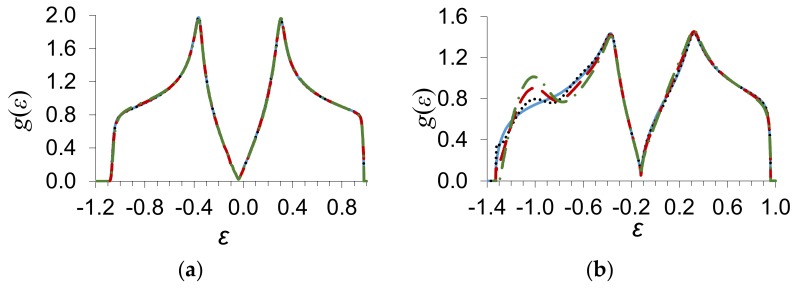
Dependence of the density of electron states $g\left(\varepsilon \right)$ on the energy ε at the concentration of a substitutional impurity y=0.2, for different values of the parameter of binary interatomic correlations on the first coordinate sphere $\varepsilon_{lj0i}^{BB}=\varepsilon_{}^{BB}$, η=0, and values of the scattering potential: (**a**) δ/w=−0.2, (**b**) δ/w=−0.6. The density of electron states calculated in the approximation of coherent potential is shown by a continuous curve with regard to the processes of scattering on the pairs of atoms in the limits of the first coordinate sphere; the dotted curve corresponds to $\varepsilon_{}^{BB}=0$, the dashed line to $\varepsilon_{}^{BB}=-0.05$, and the dash-dotted curve to $\varepsilon_{}^{BB}=-0.1$.

In Figure 1, we present the results of numerical calculations of the density of states of graphene *g*(*ε*) by Equation (9) at the substitutional impurity concentration y=0.2 and different values of the scattering potential [δ/w=−0.2 (a), δ/w=−0.6 (b)] and the parameter of binary interatomic correlations on the first coordinate sphere. The energy is given in units of the energy zone half-width w.

It is seen from Figure 1b that the appearance of the interatomic correlations is accompanied by the appearance of a characteristic burst on the curve of the energy dependence of the density of electron states. Its value increases with the parameter of correlations $\varepsilon_{}^{BB}$. The curves describing the density of electron states in the approximation of coherent potential with regard to the processes of scattering on the pairs of atoms in Figure 1a practically coincide for small values of the scattering parameter δ/w=−0.2.

In Figure 1b, the continuous curve describes the density of electron states *g*(*ε*) of graphene, which is calculated in the approximation of coherent potential according to the first term in Equation (9). The dotted curve gives the density of states *g*(*ε*) calculated with regard to the processes of scattering on the pairs of atoms located in the limits of the first coordinate sphere in the case of the completely disordered arrangement of impurity atoms, $\varepsilon_{}^{BB}=0$, η=0. The values of the density of states *g*(*ε*), calculated with regard to the processes of scattering on the pairs of atoms in the limits of three coordinate spheres and in the limits of ten coordinate spheres, practically coincide with the results of calculations that consider the scattering on the pairs in the limits of the first coordinate sphere. Thus, we may conclude that the domain of electron states of an impurity in graphene in the model under study is spatially bounded by the first coordinate sphere. 

**Figure 2:**
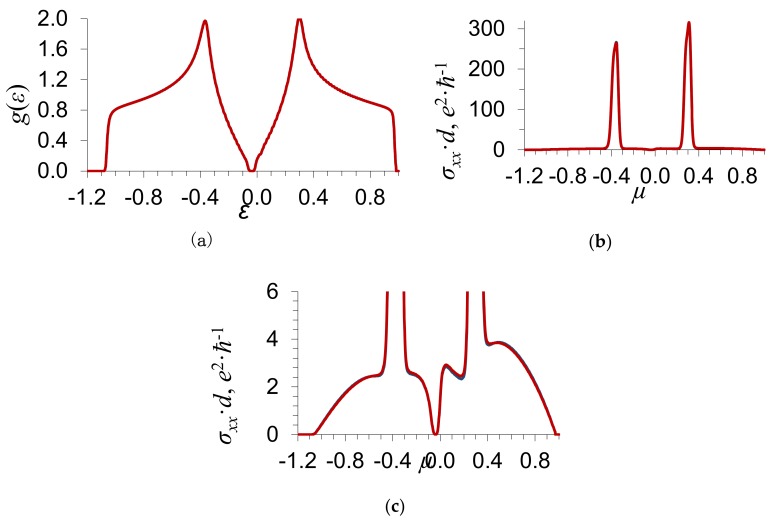
Dependence of (**a**) the density of electron states *g*(*ε*) on the energy *ε*; (**b**) the electrical conductance *σ_xx_*(*μ*) on the Fermi level *μ*, where *d* is the thickness of a graphene layer; and (**c**) the electrical conductance *σ_xx_*(*μ*) on the Fermi level *μ* (shown on a larger scale). The substitutional impurity concentration y=0.2, the scattering potential δ/w=−0.2, the order parameter η=0.3, and the parameter of binary interatomic correlations $\varepsilon_{}^{BB}=0$. The blue curve shows the results of calculations in the approximation of coherent potential, while the red curve shows those with regard to the processes of the scattering of electrons on the pairs of atoms of the first coordinate sphere.

**Figure 3:**
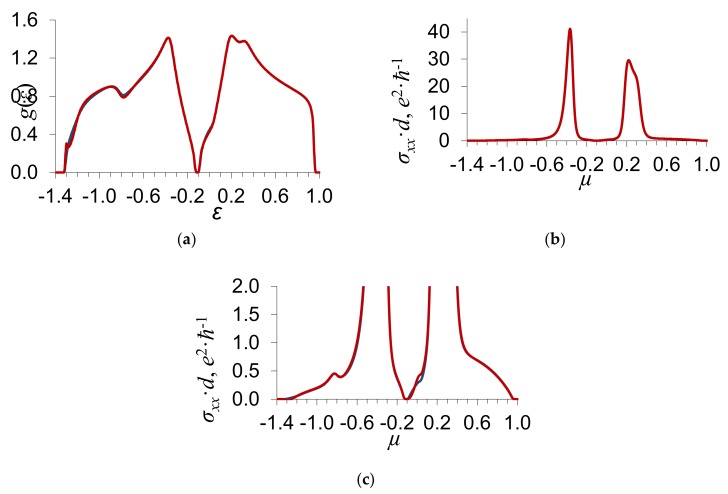
Dependence of (**a**) the density of electron states *g*(*ε*) on the energy *ε*; (**b**) the electrical conductance *σ_xx_*(*μ*) on the Fermi level *μ*, where *d* is the thickness of a graphene layer; and (**c**) the electrical conductance *σ_xx_*(*μ*) on the Fermi level *μ* (shown on a larger scale). The substitutional impurity concentration y=0.2, the scattering potential δ/w=−0.6, the order parameter η=0.3, and the parameter of binary interatomic correlations $\varepsilon_{}^{BB}=0$. The blue curve shows the results of calculations in the approximation of the coherent potential, while the red curve shows those with regard to the processes of the scattering of electrons on the pairs of atoms of the first coordinate sphere.

In Figure 2 and Figure 3, we show the density of electron states *g*(*ε*) and the electrical conductance *σ_xx_*(*μ*) as functions of the energy *ε* and the Fermi level *μ*, respectively; *d* means the thickness of a graphene layer. The calculations of *g*(*ε*) and *σ_xx_*(*μ*) are carried out by Equations (9) and (10). The values of the energy *ε* and the Fermi level *μ* are given in units of the energy zone half-width w. The substitutional impurity concentration y=0.2, the order parameter η=0.3, the parameter of binary interatomic correlations $\varepsilon_{}^{BB}=0$, the scattering potential δ/w=−0.2 (Figure 2), and δ/w=−0.6 (Figure 3). 

Figure 2 and Figure 3 indicate that, at the ordering of impurity atoms, there appears a gap in the energy spectrum of graphene in which the density of states *g*(*ε*) = 0 (Figure 2a). The electrical conductance of graphene *σ_xx_*(*μ*) for the Fermi level located in the gap is equal to zero. For the Fermi level outside the gap, the electrical conductance of graphene is nonzero and increases with the density of states on the Fermi level.

As distinct from the above-described limiting case of weak scattering $\left|\delta /w\right|\ll 1$, where the gap width increases with the scattering potential δ, the dependence of the gap width on δ in the case of strong scattering has a more complex character. As the absolute value of the scattering potential increases from $\left|\delta /w\right|=0.2$ to $\left|\delta /w\right|=0.6$, the gap width decreases a little. The dependences of the electrical conductance on the scattering potential δ and the order parameter η also have a more complicated character than in the limiting case of weak scattering $\left|\delta /w\right|\ll 1$ [14].

**Figure 4:**
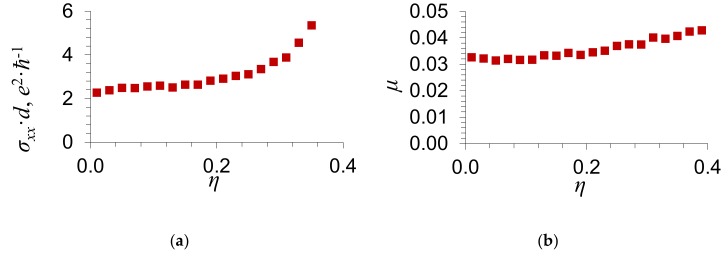

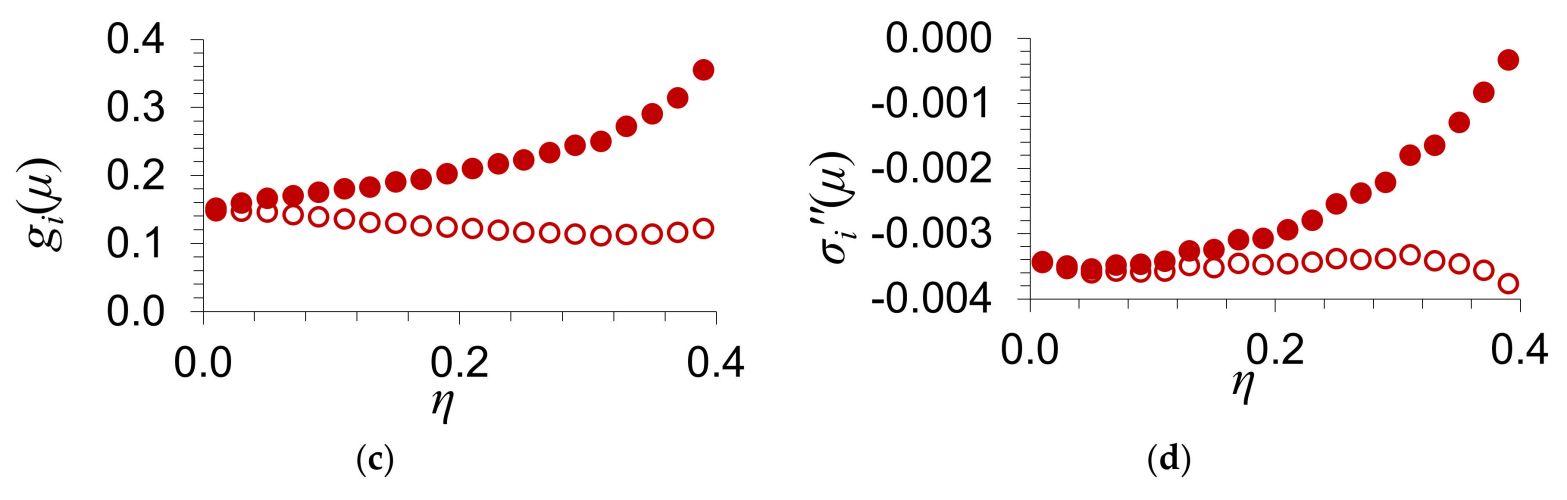
Dependence of (**a**) the electrical conductance of graphene *σ_xx_*; (**b**) the Fermi level *μ*; (**с**) the partial density of states $g_{i}\left(\mu \right)$ at the Fermi level; and (**d**) the imaginary part of the coherent potential $\sigma_{i}^{\prime \prime }\left(\mu \right)$ at the Fermi level on the order parameter of impurity atoms *η*. The substitutional impurity concentration y=0.2 and the scattering potential δ/w=−0.2. Circles correspond to $g_{1}\left(\mu \right)$ and $\sigma_{1}^{\prime \prime }\left(\mu \right)$ of the first sublattice, in which the impurity atoms are located in the case of full order. Filled circles show $g_{2}\left(\mu \right)$ and $\sigma_{2}^{\prime \prime }\left(\mu \right)$ for the second sublattice.

**Figure 5:**
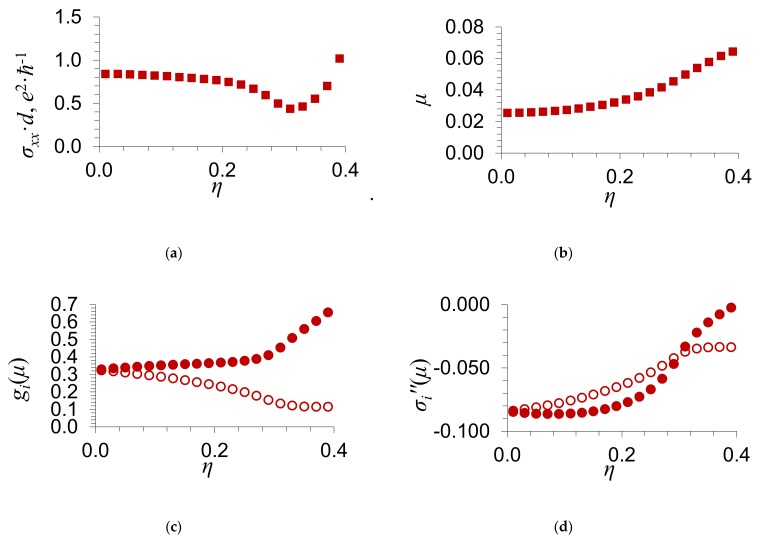
Dependence of (**a**) the electrical conductance of graphene *σ_xx_*; (**b**) the Fermi level *μ*; (**с**) the partial density of states $g_{i}\left(\mu \right)$ at the Fermi level; and (**d**) the imaginary part of the coherent potential $\sigma_{i}^{\prime \prime }\left(\mu \right)$ at the Fermi level on the order parameter of impurity atoms *η*. The substitutional impurity concentration y=0.2 and the scattering potential δ/w=−0.6. Circles correspond to $g_{1}\left(\mu \right)$ and $\sigma_{1}^{\prime \prime }\left(\mu \right)$ of the first sublattice, in which the impurity atoms are located in the case of full order. Filled circles show $g_{2}\left(\mu \right)$ and $\sigma_{2}^{\prime \prime }\left(\mu \right)$ for the second sublattice.

With the purpose to clarify the character of the dependence of the electrical conductance *σ_xx_* on the scattering potential δ and the order parameter *η*, we present the dependence of the electrical conductance of graphene *σ_xx_* on the order parameter of impurity atoms *η* for different values of the scattering potential δ in Figure 4 and Figure 5. The number of electrons per atom, whose energies are in the energy zone, is equal to 〈Z〉=1.01. For such value of 〈Z〉, the Fermi level *μ*(*η*) calculated by Equation (14) lies to the right of the energy gap. In Figure 4b and Figure 5b, we show the Fermi level *μ*(*η*) as a function of the order parameter of an impurity *η*. In Figure 4c and Figure 5c, we give the dependence of the partial density of states *g_i_*(*μ*) at the Fermi level on the order parameter of an impurity *η*; *i* = 1, 2 is the number of a sublattice. Figure 4d and Figure 5d present the dependence of the imaginary part of the coherent potential $\sigma_{i}^{\prime \prime }\left(\mu \right)$ at the Fermi level on the order parameter of impurity atoms *η*. 

It is seen from Figure 4 and Figure 5 that the electrical conductance of graphene increases with the order parameter of an impurity *η*. 

The numerical results presented in Figure 4 and Figure 5 for the electrical conductance *σ_xx_* agree qualitatively with the formula obtained in the limiting case of weak scattering $\left|\delta /w\right|\ll 1$ [14] and with Equation (25). In this case, we took into account that the dependence of the electrical conductance of graphene, which was obtained in the limiting case of weak scattering [14], on the ordering of an admixture is applicable only in the case of such concentration at which the Fermi level lies in the vicinity of the Dirac point. In the present work, we give the results of numerical calculations of the dependence of the electrical conductance of graphene *σ_xx_* on the ordering parameter *η*. We have considered such values of concentrations and scattering potentials, at which the Fermi level lies in the vicinity of the Dirac point, as well as outside it. 

As the order parameter *η* tends to the maximum value, $\eta \to \eta_{max}$, the electrical conductance $\sigma_{xx}\to \infty$. As is seen from Equation (25), this is caused by an increase in the density of states at the Fermi level $g_{2}\left(\mu \right)$ and in the relaxation time with the order parameter η [as $\eta \to \eta_{max}$, the imaginary part of the coherent potential $\sigma_{2}^{\prime \prime }\left(\mu \right)\to 0$].

## 4. Conclusions

We note that values of the density of states and the electrical conductance of graphene cannot be used, if the Fermi level falls in the interval (Equation (23)) of energies at the gap edges. The values of the density of states *g*(*ε*) calculated with regard to the processes of scattering on the pairs of atoms located in the limits of three coordinate spheres and in the limits of ten coordinate spheres practically coincide with the results of calculations involving the scattering on the pairs located in the limits of the first coordinate sphere. Thus, we may conclude that the domain of electron states of the impurity in graphene in the model under consideration is spatially bounded by the first coordinate sphere. We showed the existence of domains with localized extrinsic states on the edges of the energy gap arising at the ordering of atoms of the admixture. If the Fermi level falls in the indicated domains, the processes of the scattering of electrons on clusters consisting of at least two atoms contribute essentially to the electrical conductance of graphene, and the approximation of coherent potential fails in this case.

We have established that, at the ordering of impurity atoms, a gap appears in the energy spectrum of graphene. Its width depends on the order parameter *η* and the difference in the scattering potentials δ of an impurity atom and a carbon atom.

In the limiting case of weak scattering $\left|\delta /w\right|\ll 1$, we have shown that the gap arising at the ordering of impurity atoms in the energy spectrum of graphene has a width of $\eta \left|\delta \right|$ and is centered at the point yδ, where w is the energy zone half-width of pure graphene. 

At the electron concentration 〈Z〉 (Equation (14)), when the Fermi level falls in the arising gap, the electrical conductance tends to zero at the ordering of an impurity, $\sigma_{xx}\to 0$, i.e., a metal–dielectric transition arises.

If the Fermi level lies outside the gap, the electrical conductance *σ_xx_* increases with the order parameter η and tends to infinity, as the order parameter $\eta \to \eta_{max}$. 

An increase in the electrical conductance *σ_xx_* of graphene with the order parameter *η* of impurity atoms is caused by an increase in the density of states at the Fermi level, $g\left(\mu \right)$, and by an increase in the relaxation time of electron states, $\tau \left(\mu \right)$, tending to infinity, as $\eta \to \eta_{max}$.
